# Waller’s Case of Unavoidable Hemorrhage

**Published:** 1848

**Authors:** 


### ARTICLE IV.

### *Case of unavoidable Hemorrhage—Successful Operation of Transfusion.* By Dr. Waller.

The following interesting case occurred in the practice of Mr. Greaves. Hemorrhage appeared to an alarming extent in the eighth month. The patient appearing in a desperate state, Dr. Waller's assistance was requested. He found her in a very unpromising state, with a completely blanched countenance, pale and livid lips, cold extremities, laborious respiration, and a pulse scarcely perceptible; the general surface of the body was also cold. In short, every thing indicated approaching dissolution. Stimulants had been freely given, but they failed to excite even a temporary rally. The vagina was filled with coagula; and, as the hemorrhage appeared to haveceased, he did not thinkit expedient to disturb the clots in attempting delivery. Stimulants were again had recourse to, but with no better effect. The symptoms of exhaustion increased, and nothing but transfusion seemed, under these circumstances, to hold out the slightest chance of relief. Mr. Greaves concurring in this opinion, preparation was made for its performance. The first intention was to have laid bare the vein, and to have had all things in readiness, then to have delivered, and, provided there had been no improvement in the condition of the patient, to have transfused immediately afterwards. A little reflection convinced the operators that this plan would be fraught with danger; for, had syncope occurred, in all probability it would have been fatal. The operation was, therefore, at once commenced. When about five ounces of blood had been introduced, the amendment was evident; the pulse was more perceptible, and the countenance assumed a somewhat better aspect. The blood now flowed very sluggishly from the arm of the female who supplied it; it was, therefore, determined to wait awhile and watch the effects, nourishment and stimuli being administered occasionally. The rally continued for about two hours and a half, when the female again began to sink, and jactitation supervened; gruel, with brandy, was given without any benefit; the pulse again was just but perceptible, and the body getting cold. Dr. Waller again injected about four ounces of blood from the same individual who had previously supplied it; but this time the symptoms did not improve. The stream issuing from the punctured arm was so languid that it was not thought right to proceed, and a fresh subject was sought to furnish them with a better supply. The husband of the patient, being in the room, came forward to their aid; he looked rather pale, and, therefore, they gave him a glass of hot spirits and water, and then opened a vein, from which the blood flowed in an impetuous stream. The first injection of about two ounces produced a marked alteration in the pulse; it became decidedly perceptible. When nine ounces had been injected, the countenance was much improved; there was even a slight appearance of color in the cheeks, and pain in the arm was complained of. Four ounces more were introduced, when all symptoms of immediate danger vanished. There was no faintness afterwards; the surface was warm; the pulse steady, about 100 in the minute; jactitation ceased; and nourishment was retained on the stomach. The only complaint was of excessive fatigue, with an inclination for sleep; there were also a few "grinding pains." Dr. Waller visited her again in about an hour and a half. She had been dozing, and was extremely tranquil; reaction was perfect, and there was no hemorrhage, no tumult in the circulation. He now left the case in the hands of Mr. Greaves, who afterwards informed him that, after a sleep of some hours, the pain increased, and he felt a portion of detached placenta in the vagina; this was expelled by the natural efforts. A dead child soon followed, the remainder of the placenta coming away an hour afterwards, without hemorrhage. The mother recovered.

[The failure of the second injection from the original person to rouse the patient when the "stream issuing from the punctured arm was languid," and the instantaneous success derived from the freely flowing blood from the husband's arm, are suggestive of valuable propositions, which should not be lost sight of in practice.]

